# Fully quantitative pixel-wise analysis of cardiovascular magnetic resonance perfusion improves discrimination of dark rim artifact from perfusion defects associated with epicardial coronary stenosis

**DOI:** 10.1186/s12968-018-0436-0

**Published:** 2018-03-08

**Authors:** Allison D. Ta, Li-Yueh Hsu, Hannah M. Conn, Susanne Winkler, Anders M. Greve, Sujata M. Shanbhag, Marcus Y. Chen, W. Patricia Bandettini, Andrew E. Arai

**Affiliations:** 1 National Heart, Lung and Blood Institute, National Institutes of Health, Department of Health and Human Services, Bldg 10, Rm B1D416, MSC 1061, 10 Center Drive, Bethesda, MD 20892-1061 USA; 2 Duke University School of Medicine, Durham, North Carolina USA; 3 Medical University of Vienna, Vienna, Austria

**Keywords:** Myocardial perfusion, Dark-rim artifact, MRI, Coronary artery disease, Quantitative perfusion

## Abstract

**Background:**

Dark rim artifacts in first-pass cardiovascular magnetic resonance (CMR) perfusion images can mimic perfusion defects and affect diagnostic accuracy for coronary artery disease (CAD). We evaluated whether *quantitative* myocardial blood flow (MBF) can differentiate dark rim artifacts from true perfusion defects in CMR perfusion.

**Methods:**

Regadenoson perfusion CMR was performed at 1.5 T in 76 patients. Significant CAD was defined by quantitative invasive coronary angiography (QCA) ≥ 50% diameter stenosis. Non-significant CAD (NonCAD) was defined as stenosis by QCA < 50% diameter stenosis or computed tomographic coronary angiography (CTA) < 30% in all major epicardial arteries. Dark rim artifacts had study specific and guideline-based definitions for comparison purposes. MBF was quantified at the pixel-level and sector-level.

**Results:**

In a NonCAD subgroup with dark rim artifacts, stress MBF was lower in the subendocardial than midmyocardial and epicardial layers (2.17 ± 0.61 vs. 3.06 ± 0.75 vs. 3.24 ± 0.80 mL/min/g, both *p* < 0.001) and was also 30% lower than in remote regions (2.17 ± 0.61 vs. 2.83 ± 0.67 mL/min/g, *p* < 0.001). However, subendocardial stress MBF in dark rim artifacts was 37–56% higher than in true perfusion defects (2.17 ± 0.61 vs. 0.95 ± 0.43 mL/min/g, *p* < 0.001). Absolute stress MBF differentiated CAD from NonCAD with an accuracy ranging from 86 to 89% (all *p* < 0.001) using pixel-level analyses. Similar results were seen at a sector level.

**Conclusion:**

Quantitative stress MBF is lower in dark rim artifacts than remote myocardium but significantly higher than in true perfusion defects. If confirmed in larger series, this approach may aid the interpretation of clinical stress perfusion exams.

**Trial registration:**

ClinicalTrials.gov Identifier: NCT00027170; first posted 11/28/2001; updated 11/27/2017.

## Background

Stress tests remain an attractive approach for diagnosing coronary artery disease (CAD) [1]. Stress cardiovascular magnetic resonance (CMR) perfusion can detect CAD and additionally quantification improves the objectivity of interpretation [2].

A dark rim artifact that mimics a true defect is one important limitation in CMR perfusion. There are numerous causes of dark rim artifact including: Gibbs ringing [3], inadequate spatial resolution [4, 5], motion artifacts [6], non-uniformity across k-space [7], and partial volume errors. Higher heart rates can exacerbate cardiac motion creating larger dark rim artifacts [8]. To complicate matters, different artifacts from multiple causes can combine to exacerbate dark rim artifact.

Quantitative measurements of myocardial blood flow (MBF) might provide insight into differentiating dark rim artifacts from true perfusion defects. Our primary aim was to use fully quantitative analysis of perfusion CMR at the pixel-level to characterize dark rim artifacts, normal perfusion, and true perfusion defects. We also aimed to understand how well qualitative assessment of dark rim artifacts published by the Society for Cardiovascular Magnetic Resonance (SCMR) guidelines [9] differentiated CAD from those without CAD (NonCAD).

## Methods

### Patient population

The aim to characterize dark rim artifacts was a retrospective design we felt that could be addressed with the use of existing data. All CMR scans were prospectively acquired from under a research clinical trial (ClinicalTrials.gov Identifier: NCT00027170) but subjects were retrospectively selected for this analysis of dark rim artifacts. The clinical trial was approved by the institutional review board. All subjects gave written informed consent. Prospective exclusion criteria included contraindication to regadenoson, unstable angina within 48 h, hemodynamic instability, acute renal failure, estimated glomerular filtration rate < 30 ml/min/1.73m^2^, claustrophobia, and certain metallic implants. Patients were referred to assess either a chest pain equivalent syndrome, had a prior equivocal nuclear stress test, or to assess the significance of a known coronary stenosis.

**Fig. 1:**
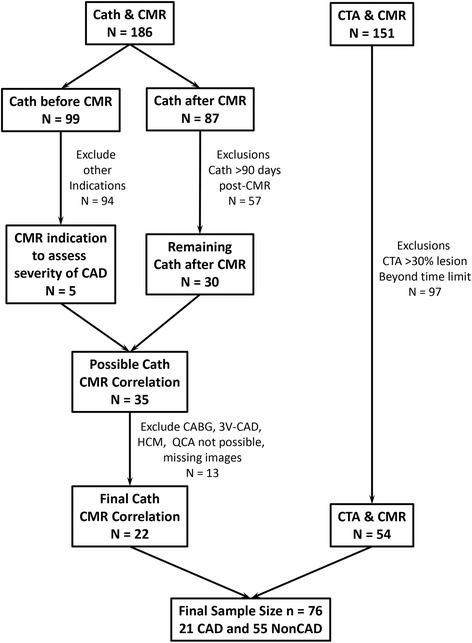
Flow diagram summarizing selection of study participants for the study

For this retrospective analysis of dark rim artifacts, studies were selected by finding the overlap between three clinical research databases summarizing stress perfusion CMR studies, Computed tomography (CT) coronary angiography studies, and invasive coronary angiography (CATH) exams (Fig. 1). Consecutive patients over a 1.3 year period (2010–2012) with a quantitative regadenoson perfusion CMR exam and correlative quantitative invasive coronary angiography (QCA) or CT coronary angiogram (CTA) within 90 days of CMR were included in the retrospective analysis. Patients with coronary artery bypass, congenital heart disease, significant co-morbid conditions, or technical issues with the CMR acquisition were excluded. Patients with severe three-vessel CAD (QCA ≥ 70% stenosis in all three vessels) were also excluded by study design as we aimed to compare MBF in abnormal regions relative to the remote myocardium. NonCAD patients were excluded if they had a history of percutaneous coronary intervention or myocardial infarction.

### Standard of reference

Significant CAD was defined by an intermediate stenosis (50–69% diameter) or severe stenosis (≥ 70% diameter) in any major coronary artery by QCA. Invasive QCA (Artis, Siemens AG, Berlin, Germany) was measured by a physician blinded to the clinical history and CMR perfusion results. NonCAD was defined by invasive QCA (< 50% diameter) or CTA stenosis (< 30% diameter). The attribution of vessel territories to each perfusion defect was performed unblinded after the QCA.

### Vasodilator stress CMR perfusion

The CMR exam was performed on a 1.5 T scanner (Magnetom Avanto or Magnetom Espree, Siemens Healthineers, Erlangen, Germany) using a balanced steady state free procession (bSSFP) sequence with a ‘dual sequence’ technique to measure the arterial input function [10]. Three myocardial slice locations were imaged during every R-R interval over 60 heartbeats. Typical imaging parameters included: a three 90° sequel preparation pulse, 50° readout flip angle, 95 ms saturation recovery time, a 2.3 ms repetition time, a 1.22 ms echo time, a typical field of view of 360x270mm^2^, an acquisition matrix of 128 × 96, an image matrix of 256 × 192 after interpolation, and 8 mm slice thickness. Parallel imaging was used with an acceleration factor of 2. Non-rigid motion correction was used to facilitate pixel-wise perfusion quantification.

Antianginal medications were held for the CMR scan and patients were instructed to avoid caffeine for 24 h. Stress perfusion imaging was performed 70 s after the 400 mcg bolus of regadenoson and rest imaging occurred 20 min later. Gadolinium-DTPA (Magnevist, Berlex Laboratories, Wayne, New Jersey, USA; 0.05 mmol/kg) was injected during the stress and rest first pass perfusion imaging at 5 ml/s.

### Myocardial blood flow quantification and comparison

Quantitative pixel maps of perfusion were created as previously described [11] with a few refinements over those methods. The perfusion images had automated motion correction from the scanner. The user still needed to draw epicardial contours, endocardial contours, and select a most normal appearing myocardial region. The amount of labor involved was greatly reduced from prior methods. The program used the same fundamental quantification steps but would process a circular field of view larger than the left ventricular (LV) myocardium. Results were displayed such that each myocardial pixel shows the estimated MBF value in units of mL/min/g.

Because the appearance of dark rim artifacts and true perfusion defects can be narrow, regional MBF were measured from the perfusion pixel maps by 3-layers: subendocardial, midmyocardial, and epicardial layers. Regions of interest in different layers were sized based on the extent of the dark rim artifact and perfusion defects. Sector level analysis was also performed by dividing the myocardium into 6 segments then dividing each segment into inner and outer sub-sectors.

### Definitions of dark rim artifacts

Since this paper aimed to examine the characteristics of dark rim artifacts and whether they might explain some false positive perfusion defects, we defined a dark rim artifact as hypointense subendocardial regions on stress perfusion images in the NonCAD group. This definition is different from prior definitions, but benefits from the prior knowledge that this group of subjects cannot have true positive perfusion defects.

We also studied the diagnostic performance of dark rim artifact definitions as described in the SCMR Guidelines [9] which have three definitions of dark rim artifact: 1) An apparent perfusion defect seen in stress that lasts < 7 beats is considered a “dark rim artifact” while one that lasts ≥ 7 beats is called a “true positive perfusion defect,” 2) An apparent perfusion defect seen at rest and at stress is a “dark rim artifact” while one at stress only is a “true positive defect,” and 3) if myocardial signal intensity decreases below baseline prior to myocardial enhancement, the apparent defect is a dark rim artifact.

It is important to recognize this paper’s specific focus in quantitative analysis goes beyond the SCMR guidelines [9]. We used the SCMR definition of a dark rim artifact only in qualitative diagnostic accuracy analyses of those specific criteria, while our quantitative MBF comparison was not restricted to those criteria.

### Statistical analysis

For quantitative comparison of MBF measurements in dark rim artifact vs. true perfusion defects, the patients were divided into two distinct groups: CAD defined by QCA ≥ 50% stenosis versus NonCAD subgroup with DRA.

All patients were analyzed using standard contingency tables, sensitivity, specificity, accuracy, positive predictive value, negative predictive value, and area under the curve. MedCalc (version 12.7.7.0, Mariakerke, Belgium) was used for statistical comparisons. Values were expressed as mean ± SD. The Kolmogorov-Smirnov test was used to test for Normal distribution. The Student’s *t*-test was used for pairwise comparison of normally distributed data. The Wilcoxon and Mann-Whitney tests were applied to non-normally distributed data. The Fisher’s Exact test was used to compare discrete variables. A Bonferroni correction was applied for multiple comparisons when necessary. Receiver operator characteristics curves were used to evaluate the discriminatory performance for prediction of CAD.

## Results

### Baseline characteristics of patient population

**Fig. 2:**
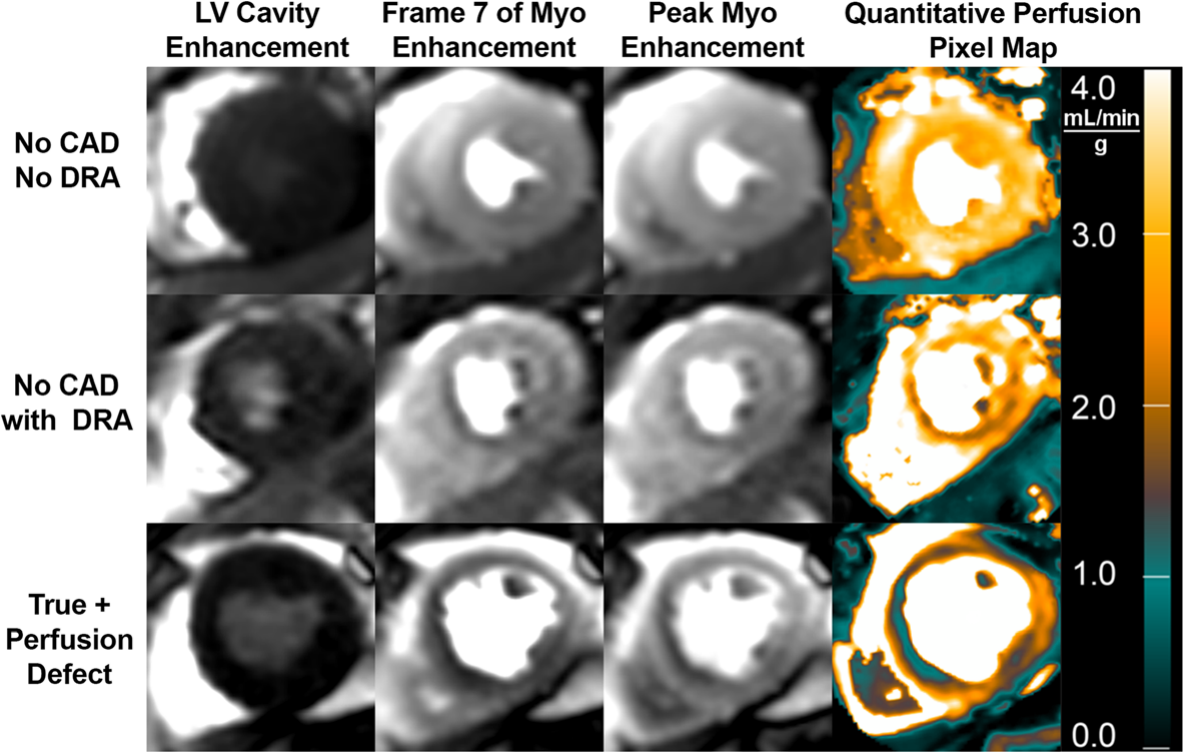
Examples of a Normal Perfusion Study, a Dark Rim Artifact, and a True Positive Perfusion Defect. The examples illustrate “dark regions” in the subendocardium of CMR perfusion images and appearances on MBF maps. Quantitative measurements of MBF in these regions differentiated the dark rim artifacts from true perfusion defects

**Fig. 3:**
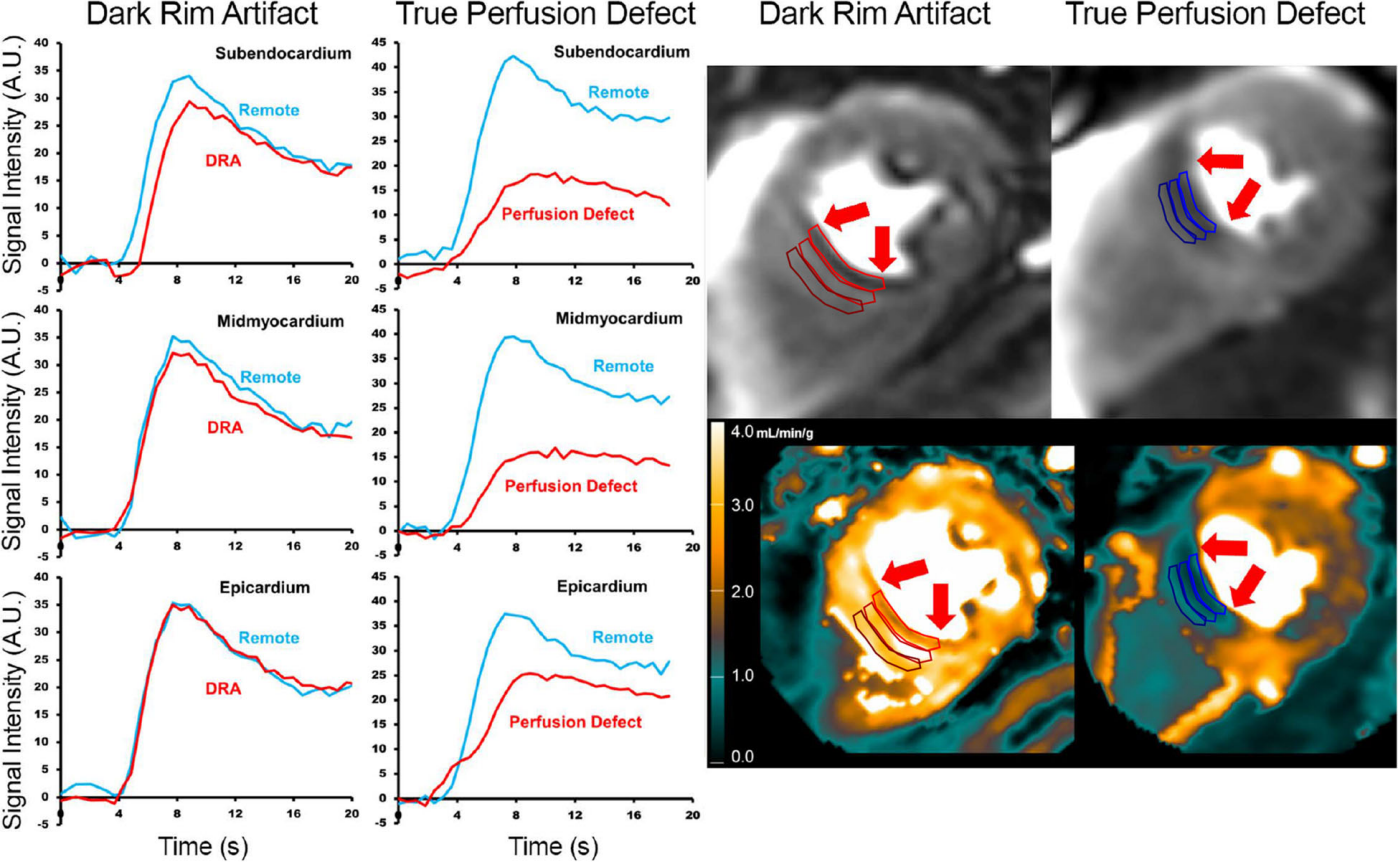
Raw Stress Perfusion CMR images and Signal Intensity Curves in Dark Rim Artifacts and True Perfusion Defect. The dark rim artifacts in the septum (arrows) has three findings: (1) a decrease in the signal intensity curve prior to contrast arrival, (2) a delay in enhancement relative to the remote zone, and (3) a lower signal intensity throughout the upslope and peak of myocardial enhancement. However, the signal intensity curves in the midmyocardium and epicardium are not affected by the dark rim artifacts. In the true perfusion defect, the amplitude, upslope and persistence of the signal intensity abnormalities are all different than the remote region

Patients (*N* = 76) averaged 55.9 ± 12.2 years of age and 55% (n = 41) were male (Table 1). Patient inclusion and exclusion is summarized in Fig. 1. On a per patient basis, 28% (*N* = 21) had significant QCA defined CAD and 18 of these 21 patients had at least one stenosis ≥ 70 by QCA (Table 2). A total of 84 coronary arteries were analyzed by QCA (left main, left anterior descending (LAD), circumflex (CX), and right coronary (RCA) arteries). There were 47 vessels with 0–49% stenosis, 13 vessels with intermediate stenosis (50–69%) and 24 vessels with severe stenosis (≥ 70%). Within the NonCAD group, a subgroup of patients (*N* = 45) were identified as having dark rim artifact. Examples of normal, dark rim artifact, and true perfusion defects are shown in Figs. 2 and 3.

**Table 1:** Demographic summary stratified by coronary artery disease status

	NonCAD (*N* = 55)		CAD (*N* = 21)		*p*-value
Cardiovascular Risk Factors					
Age	53.0 ± 11.7		63.0 ± 10.1		**0.001**
Male	29	53%	15	71%	0.195
Hypertension	26	47%	14	67%	0.199
Hyperlipidemia	26	47%	18	86%	**0.004**
Diabetes	5	9%	5	24%	0.128
Smoking	21	38%	12	57%	0.196
Family history of CAD	14	25%	10	48%	0.097
Body mass index (kg/m^2^)	29.1 ± 6.6		28.3 ± 4.6		0.81
Past Medical History					
Prior PCI	0	0%	3	14%	**0.019**
CABG^a^	0	0%	0	0%	1.000
Prior cerebrovascular accident	0	0%	1	5%	0.276
Medications					
Aspirin or Anti-Platelet	29	53%	15	71%	0.25
Beta blocker	19	35%	11	52%	0.27
Calcium channel blocker	4	7%	1	5%	0.84
Nitrate	2	4%	1	5%	0.65
Diuretic	8	15%	2	19%	0.94
ACE inhibitor	13	24%	8	38%	0.35
ARB	4	7%	2	10%	0.97
Statin	22	40%	19	90%	**< 0.001**

*Abbreviations*: *ACE* angiotensin converting enzyme inhibitor, *ARB* angiotensin receptor blocker, *BP* blood pressure, *CABG* coronary artery bypass grafting, *CAD* coronary artery disease, *LV* left ventricular, *LVEDV* left ventricular end diastolic volume, *LVESV* left ventricular end systolic volume, *LVEDMass* left ventricular end diastolic mass, *LGE* late gadolinium enhancement, *N/A* not applicable, *PCI* percutaneous coronary intervention

*p* values that were significant (*p*<0.050 or lower) are in bold

^a^Patients with a prior history of coronary artery by-pass surgery and 3 vessel CAD were excluded from this study

**Table 2:** Summary of coronary artery disease status, hemodynamics, and CMR findings

	NonCAD (*N* = 55)		CAD (*N* = 21)		*p*-value
Extent of CAD by QCA					
Left main disease	N/A		0	0%	N/A
LAD disease	N/A		16	76%	N/A
CX disease	N/A		9	43%	N/A
RCA disease	N/A		12	57%	N/A
3-vessel CAD^a^	N/A		0^a^	0%^a^	N/A
2-vessel CAD	N/A		16	76%	N/A
1-vessel CAD	N/A		5	24%	N/A
Severity of stenosis					
0–49%	N/A		47	56%	N/A
50–69%	N/A		13	15%	N/A
≥ 70%	N/A		24	29%	N/A
Hemodynamics					
Baseline systolic BP (mmHg)	131.3 ± 14.7		137.0 ± 12.7		0.122
Baseline diastolic BP (mmHg)	79.5 ± 10.7		78.3 ± 13.3		0.875
Baseline heart rate (bpm)	67.1 ± 12.1		66.7 ± 10.7		0.891
Baseline rate pressure Product (bpm^a^mmHg)	9148 ± 1784		8802 ± 1785		0.452
Stress systolic BP (mmHg)	128.8 ± 17.7		134.2 ± 16.7		0.224
Stress diastolic BP (mmHg)	74.5 ± 14.2		72.3 ± 14.5		0.548
Stress heart rate (bmp)	101.2 ± 14.0		94.7 ± 13.7		0.070
Stress rate pressure product (bmp^a^mmHg)	12,709 ± 2472		13,053 ± 2743		0.617
CMR Findings					
LV ejection fraction (%)	64.1 ± 6.5		60.3 ± 6.5		**0.047**
LV stroke volume (ml)	100.4 ± 21.7		93.1 ± 25.0		0.213
LVEDV (ml)	159.2 ± 41.8		157.0 ± 45.1		0.841
LVESV (ml)	58.7 ± 25.5		63.9 ± 29.1		0.453
LVED Mass (g)	98.0 ± 28.8		107.7 ± 25.5		0.177
LGE evidence of infarction	0	0%	8	38%	**< 0.001**

*Abbreviations*: *BP* blood pressure, *CX* circumflex coronary artery, *LGE* late gadolinium enhancement, *LV* left ventricular, *LVEDV* left ventricular end diastolic volume, *LVESV* left ventricular end systolic volume, *LVEDMass* left ventricular end diastolic mass, *LGE* late gadolinium enhancement, *N/A* not applicable

*p* values that were significant (*p*<0.050 or lower) are in bold

^a^Patients with a prior history of coronary artery by-pass surgery and 3 vessel *CAD* coronary artery disease were excluded from this study

### Stress myocardial blood flow in patients with CAD

**Fig. 4:**
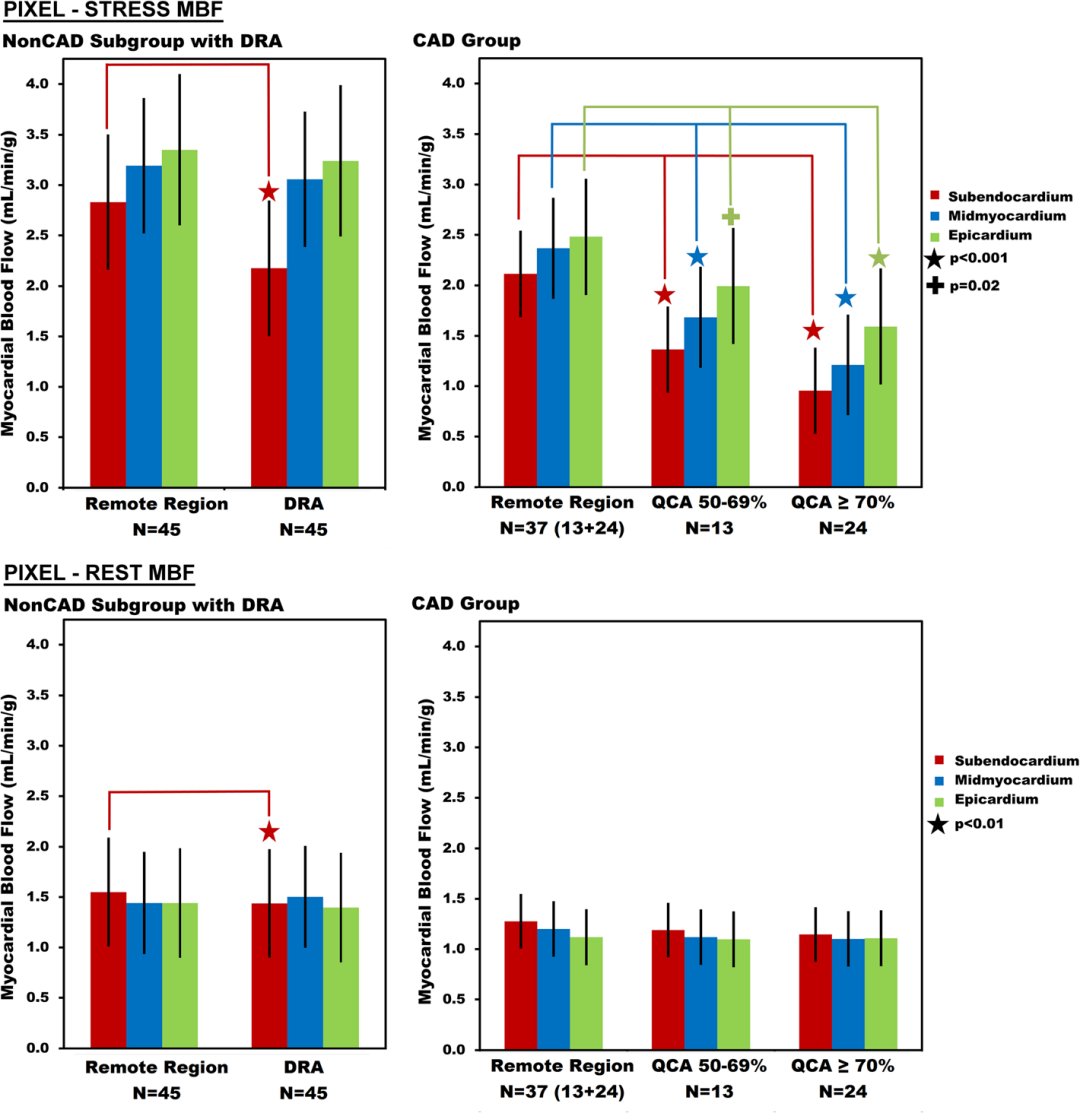
Pixel-wise Stress and Rest Myocardial Blood Flow in Remote Myocardium, Dark Rim Artifacts, Intermediate-CAD, and Severe-CAD. In the subgroup analysis of NonCAD with dark rim artifacts, the subendocardium of the dark rim artifacts region was the only layer with lower stress MBF compared to the remote myocardium. In patients with both intermediate CAD (50–69% QCA stenosis) and severe CAD (QCA ≥ 70%), stress MBF in all layers of the myocardium was lower than the corresponding remote region. Rest MBF measurements did not differentiate true positive CAD region from remote myocardium

**Fig. 5:**
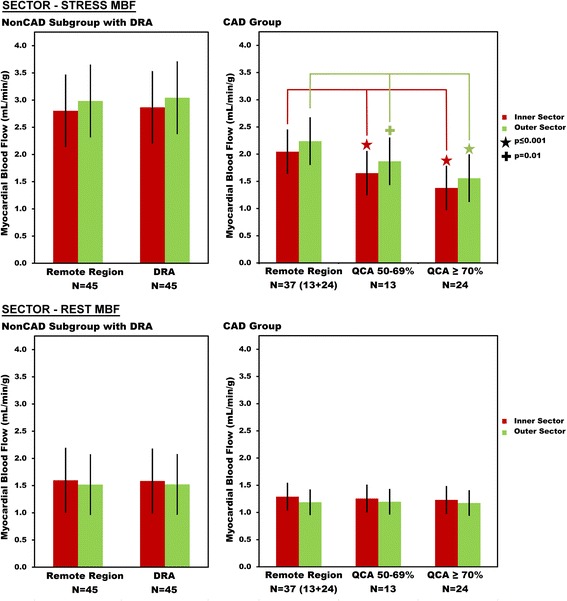
Sector-wise Stress and Rest Myocardial Blood Flow in Remote Myocardium, Dark Rim Artifacts, Intermediate-CAD, and Severe-CAD. In the subgroup of NonCAD patients with dark rim artifacts, no significant difference was found between the inner sector MBF for dark rim artifacts region compared to remote myocardium. In patients with CAD, stress MBF in both layers of the myocardium was lower than the corresponding remote region for both intermediate CAD (50–69% QCA stenosis) and severe CAD (QCA stenosis ≥ 70%)

Consistent with known subendocardial vulnerability to ischemia, there was a more severe decrease in stress MBF in the subendocardium compared with other layers in patients with CAD seen in both pixel and sector measurements (Figs. 4 and 5). In patients with either a ≥ 70% QCA stenosis or a 50–69% QCA stenosis, stress MBF was lower downstream from the stenosis in all three layers of myocardium, but most severely affected in the subendocardium (Fig. 4, all comparisons either *p* = 0.016 or *p* < 0.001). The transmural gradient of lower perfusion toward the subendocardium was most prominent in patients with severe CAD (≥ 70% QCA stenosis), but also present in patients with intermediate CAD (50–69% QCA stenosis).

### Quantitative analysis to characterize dark rim artifacts vs. perfusion defects associated with CAD

#### Pixel-wise analysis

In the NonCAD subgroup with dark rim artifacts, stress subendocardial MBF was lower in the dark rim artifact than in the remote myocardium (Fig. 4, *p* < 0.001). However, stress MBF in the midmyocardium and epicardium were not significantly different in the dark rim artifact region versus remote myocardium.

Stress subendocardial MBF in dark rim artifact in the NonCAD subgroup was significantly higher than stress MBF downstream from either a ≥ 70% QCA stenosis or a 50–69% QCA stenosis (Fig. 4, *p* < 0.001). Thus, although dark rim artifacts visually look darker and had lower MBF measurements than remote regions, quantitative MBF in these regions differentiated dark rim artifacts from perfusion defects associated with CAD (Fig. 4).

#### Sector-wise analysis

Similar to the pixel-wise quantification, sector-wise MBF measurements were able to differentiate dark rim artifacts from intermediate and severe stenosis (Fig. 5). However, there was no statistically significant difference between dark rim artifacts and the remote regions within the NonCAD subgroup (Fig. 5). Since inner sectors extended halfway across the myocardium and were frequently larger in the circumferential direction than the dark rim artifacts, these 2-layers sector-level measurements included myocardium outside of the visually apparent dark rim artifacts. Thus the 3-layers of pixel-wise analyses were smaller in both transmural and circumferential extent than the two-layer sectors.

### Implications for differentiating CAD from NonCAD

**Fig. 6:**
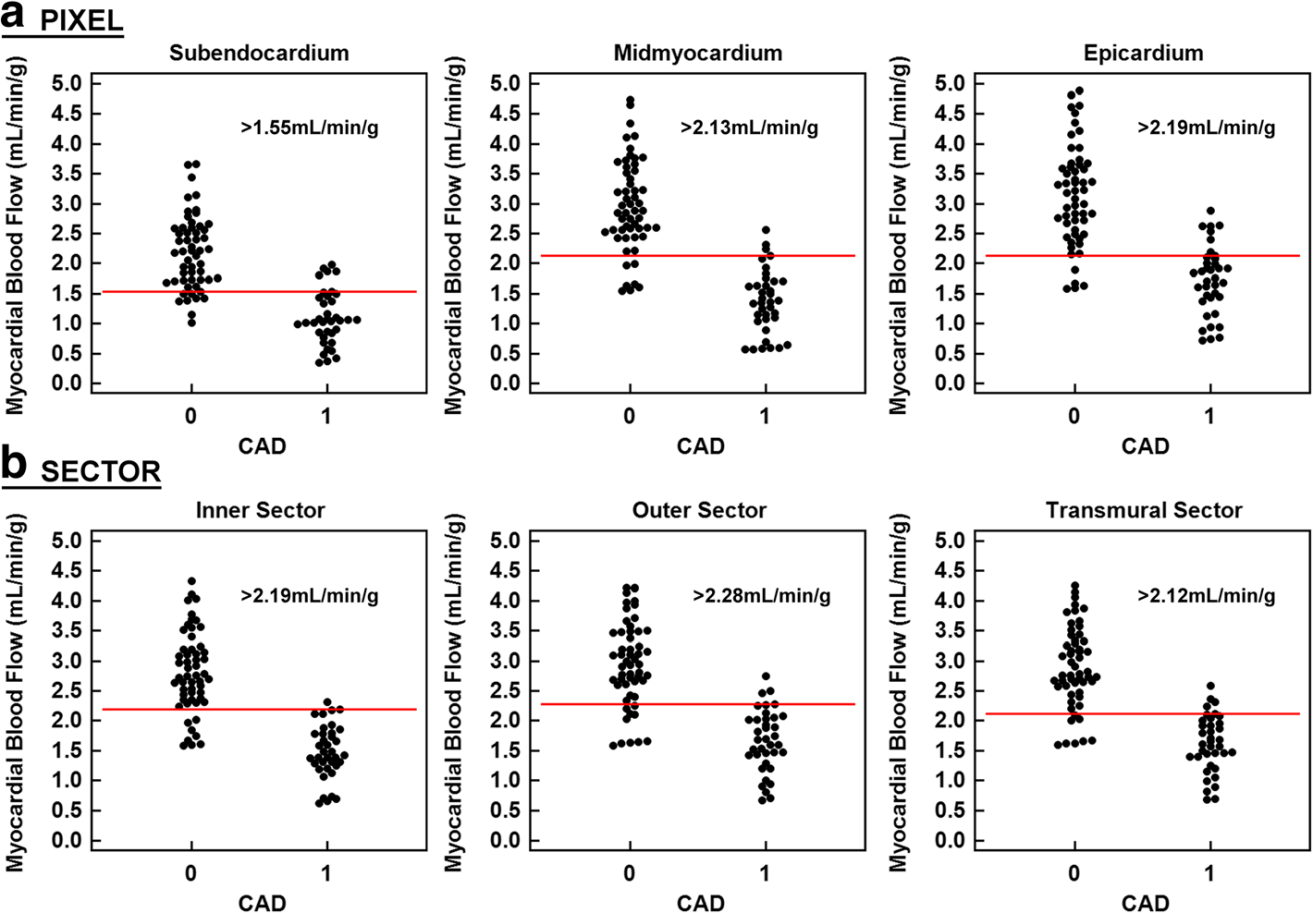
Optimal thresholds for distinguishing CAD from NonCAD at the pixel level and at the sector level for two layers and transmural sectors. Panel "**a**" represents results for the pixel-wise analysis. Panel "**b**" summarizes results for the sector-wise analysis

Using pixel-wise measurements, the group of patients with significant CAD on a per-territory basis could be differentiated from the NonCAD group by stress subendocardial MBF with an accuracy of 86% (*p* < 0.0001, Fig. 6). The sensitivity was 86% and the specificity was 85%. The stress MBF in the midmyocardium had an accuracy of 89% (*p* < 0.0001) with a sensitivity of 92% and specificity of 87%. The stress MBF in the epicardium had an accuracy of 86% (*p* < 0.001) with a sensitivity of 84% and specificity of 87%. Similar results were seen for sector-level results but with different optimal thresholds.

The optimal MBF threshold for distinguishing patients with CAD from NonCAD was 1.55 mL/min/g in the subendocardium, 2.13 mL/min/g in the midmyocardium, and 2.19 mL/min/g in the epicardium (Fig. 6). Higher thresholds apply to the sector-level analysis (Fig. 6).

### Rest myocardial blood flow

While stress MBF clearly differentiated significant CAD from remote myocardium in all layers of myocardium, the rest perfusion metrics were not able to differentiate regional MBF differences in the distribution of coronary stenoses vs. remote myocardium (*p* = NS).

### Performance of SCMR guideline advice for differentiating dark rim artifacts from true perfusion defects

Using the SCMR Guideline to define DRA persisting < 7 beats and “true perfusion defects” as ≥ 7 heartbeats had a sensitivity of 95%, but a specificity of only 18% for significant CAD. Thus, dark rim artifacts were *not* differentiated from true perfusion defects by the duration of dark region alone.

Combining an analysis of a dark region on the 7th frame of myocardial enhancement on both rest and stress perfusion images as a method of distinguishing a “perfusion defect” from a “dark rim artifact” improved the specificity to 75%, but decreased the sensitivity to 71%.

A decrease in signal intensity less than 2SD from the baseline prior to myocardial enhancement was found in only 6 of the 76 subjects. Using this measurement as an indicator of a dark rim artifact had poor diagnostic performance of a 90% sensitivity, but only a 7% specificity despite some excellent examples (Fig. 3).

### Quality assurance metrics

Three observations support that the perfusion pixel-maps can differentiate about three layers across the myocardium:

1. For subjects with significant CAD during stress perfusion, myocardial wall thickness averaged (10.4 ± 2.24 mm) which was equivalent to 3.0 ± 0.6 non-interpolated pixels across the wall and twice as many interpolated pixels.
2. At rest, subendocardial MBF was greater than epicardial MBF within the remote myocardium of both NonCAD and CAD in pixel-level (Fig. 4, *p* = 0.02 and Fig. 4, *p* = 0.002 respectively) and sector-level (*p* ≤ 0.0001 and *p* = 0.003 respectively, Fig. 5).
3. The difference between endocardial and epicardial MBF in patients with CAD was larger on the 3-layer pixel-wise comparison (Fig. 4) than the 2-layer sector-level analysis (Fig. 5). For the group of patients with a 50–69% QCA stenosis, endocardial MBF was only 12% lower than epicardial MBF on sector-level analysis, but 32% lower on pixel-level measurements. For a ≥ 70% stenosis, endocardial MBF was 12% lower than epicardial MBF on sector-level analysis but 40% lower on pixel-level measurements.

## Discussion

This is the first perfusion CMR study that specifically measured MBF at the pixel-level in dark rim artifacts and compared them to the MBF measurements in true perfusion defects in a large series of patients. In our study, fully quantitative measurements of CMR stress MBF distinguished dark rim artifacts from true perfusion defects for both intermediate (50–69% QCA stenosis) and severe CAD (≥ 70% QCA stenosis). MBF measures in dark rim artifacts, despite being lower than remote myocardium, were quantitatively *less* severely decreased than true perfusion defects. From our perfusion pixel map analysis, dark rim artifacts typically did not extend into the midmyocardium or epicardium while true perfusion defects did. We found that measurable dark rim artifact frequently lasted longer than 7 heart beats after myocardial contrast arrival indicating that the SCMR recommended criterion for differentiating a dark rim artifact from “true perfusion defects” associated with CAD is not sufficiently specific.

Dark rim artifacts could be one factor affecting the diagnostic accuracy of CMR perfusion trials. A dark rim artifact that mimics a subendocardial perfusion defect can lead to a false positive diagnosis of CAD. Conversely, minor perfusion defects may be underdiagnosed if they resemble a dark rim artifact. Thus, dark rim artifacts could adversely affect both specificity and sensitivity of stress CMR. From a recent meta-analysis, the diagnostic performance of CMR was comparable to single photon emission computed tomography (SPECT) and positron emission tomography (PET) [12]. However, 14 of 27 CMR stress perfusion studies had specificity < 80% and the multicenter MR-IMPACT II study had a sensitivity of 67% and specificity 61% [13]. On the other hand, the large CE-MARC study had higher sensitivity (86.5%) and specificity (83.4%) [14]. Thus, expert readers can interpret stress perfusion studies with very good sensitivity and specificity and must have learned how to differentiate true perfusion defects from dark rim artifacts. For example, Greenwood et al. was able to reduce unnecessary invasive coronary angiography from 28.8% in the NICE Guideline Group to 7.5% in the CMR group. [15]

The prevalence of dark rim artifacts on CMR perfusion imaging has been reported to range from (55–100%) depending on the imaging technique and the method used to identify a dark rim artifacts [16, 17]. In our study, the NonCAD cohort had an 82% prevalence of hypointense subendocardial regions on stress perfusion images. While this prevalence was on the high end, a bSSFP sequence tends to cause more dark rim artifactsthan other CMR methods. The apparent severity of dark rim artifacts is also dependent on the contrast and brightness used to display gray scale perfusion images.

A number of acquisition improvements have been developed to reduce dark rim artifacts, including k-t SENSE accelerated parallel imaging [5], increased spatial resolution [7], non-Cartesian imaging [18, 19], and filtering [4]. Since dark rim artifacts have not been eliminated, alternative strategies are being explored.

Since eliminating dark rim artifacts has been difficult in CMR perfusion, there is a need for an objective, sensitive, and specific technique to differentiate true CAD from artifacts. Semi-quantitative MBF from CMR can perform comparably with other perfusion reference standards such as SPECT and PET for diagnostic studies [14, 20, 21]. However, semi-quantitative methods tend to underestimate MBF which reduces the ability to differentiate normal from abnormal MBF [22].

In recent studies, quantitative CMR stress MBF outperformed qualitative and semi-quantitative methods [23]. Fully quantitative measurements more effectively described the extent of perfusion defects [24]. Quantification at the pixel level has been validated in canines by microspheres [11], in phantoms [25], and in humans by PET [26]. Pixel-wise quantification of MBF provides high resolution and may improve diagnostic accuracy.

### Limitations

The limitations of this study include the lack of fractional flow reserve (FFR) as a comparison, the small single center design, and the lack of an independent validation dataset. The correlation between a vessel’s physiologic significance and the percent stenosis measured by QCA is imperfect. However, QCA has been used commonly in studies evaluating the diagnostic performance of non-invasive stress testing. Fractional flow reserve wasn’t available in most of the patients in this study. Use of CTA in the inclusion/exclusion criteria may be questioned but was justified by the high negative predictive value of the test. Use of CTA also helps reduce referral bias compared with studies that recruit patients scheduled for invasive coronary angiography and better represents the population undergoing stress tests.

The lack of an independent validation set mean these results should be confirmed in an independent dataset. Until such a study is performed, the thresholds depicted should be considered conceptually important (i.e. dark rim artifacts appear to have MBF between normal perfusion and perfusion defects associated with significant CAD). The absolute thresholds may not be applicable to other perfusion methods.

The bSSFP perfusion sequence has a higher contrast to noise ratio than other methods, but it may increase the appearance of dark rim artifacts causing more false positives. Further study is needed to address whether our findings are translatable to other methods used in CMR perfusion imaging.

Currently, there is no other technique to measure microvascular flow in dark rim artifact regions to evaluate whether dark rim artifacts represent a microvascular perfusion defect or an artifact. Invasive measurements of microvascular perfusion indices were not available in any patients in this study. Thus, some fraction of dark rim artifacts could represent real microvascular disease.

## Conclusion

The ability to objectively differentiate between artifact and true perfusion defect potentially represents an important breakthrough for a common problem that affects confidence in interpretation of CMR perfusion. Larger prospective studies are needed to confirm our findings.

## Abbreviations

BP: Blood pressure
bSSFP: Balanced steady state free precession
CAD: Coronary artery disease
CMR: Cardiovascular magnetic resonance
NonCAD: Patients *without* coronary artery disease
PCI: Percutaneous coronary intervention
PET: Positron emission tomography
QCA: Quantitative coronary angiography
RCA: Right coronary artery
SCMR: Society for Cardiovascular Magnetic Resonance
SPECT: Single photon emission computed tomography
